# Comparison of Two Reconstructive Techniques in the Surgical Management of Four-Level Cervical Spondylotic Myelopathy

**DOI:** 10.1155/2015/513906

**Published:** 2015-01-27

**Authors:** FengNing Li, ZhongHai Li, Xuan Huang, Zhi Chen, Fan Zhang, HongXing Shen, YiFan Kang, YinQuan Zhang, Bin Cai, TieSheng Hou

**Affiliations:** ^1^Department of Orthopedics, Third Affiliated Hospital of PLA, Second Military Medical University, Shanghai 200433, China; ^2^Department of Orthopedics, Changhai Hospital, Second Military Medical University, Shanghai 200433, China; ^3^Department of Orthopedics, First Affiliated Hospital of PLA General Hospital, Beijing 100048, China

## Abstract

To compare the clinical efficacy and radiological outcome of treating 4-level cervical spondylotic myelopathy (CSM) with either anterior cervical discectomy and fusion (ACDF) or “skip” corpectomy and fusion, 48 patients with 4-level CSM who had undergone ACDF or SCF at our hospital were analyzed retrospectively between January 2008 and June 2011. Twenty-seven patients received ACDF (Group A) and 21 patients received SCF. Japanese Orthopaedic Association (JOA) score, Neck Disability Index (NDI) score, and Cobb's angles of the fused segments and C2-7 segments were compared in the two groups. The minimum patient follow-up was 2 years. No significant differences between the groups were found in demographic and baseline disease characteristics, duration of surgery, or follow-up time. Our study demonstrates that there was no significant difference in the clinical efficacy of ACDF and SCF, but ACDF involves less intraoperative blood loss, better cervical spine alignment, and fewer postoperative complications than SCF.

## 1. Introduction

There is currently no consensus on the best surgical treatment method for multilevel cervical spondylosis myelopathy (CSM). Surgery through an anterior approach was firstly described by Robinson and Smith [1] in 1955 as a successful treatment for cervical disease. Several decades later following the development of surgical techniques and advances in internal fixation materials, anterior approach surgery has become widely accepted; however, controversy remains regarding the selection of surgical procedures for the treatment of multilevel CSM.

Surgeries using both anterior and posterior approaches have been developed with the goal of decompressing the spinal cord and restoring the stability of the cervical spine. The posterior approach involves laminectomy with or without fusion, or laminoplasty. Laminectomy and laminoplasty have been found to be effective treatment for multilevel CSM but are hindered by the complications of progressive cervical kyphosis, C5 nerve root palsy, axial neck pain, segmental instability, and associated postoperative neurological deterioration [2–5]. While the anterior approach surgery directly decompresses the spinal cord and nerve root, improves cervical alignment, and reduces the incidence of complications. Several studies have confirmed the safety and efficacy of treating multisegmental CSM using an anterior approach [6–9]. Different methods of anterior approach surgery include contiguous corpectomy and fusion (CCF), 4-level anterior cervical discectomy and fusion (ACDF) [10], “skip” corpectomy and fusion (SCF) [11], and hybrid decompression and fusion (HDF) [12]. During CCF it is necessary to remove three continuous vertebral bodies. The procedure requires long anterior strut grafts and postoperative complications include pseudarthrosis, graft subsidence, and loss of cervical lordosis [13–15]. For this reason only ACDF, SCF, and HDF continue to be widely used.

Based on these factors, the decision to treat multilevel CSM especially 4-level CSM with multilevel ACDF rather than SCF remains controversial, and few studies have investigated the treatment of 4-level CSM. In the present study, data from patients with 4-level CSM who underwent ACDF and SCF were retrospectively analyzed to compare the clinical efficacy, radiological outcomes, and complications associated with these two reconstructive techniques.

## 2. Subjects and Methods

### 2.1. Subject

Between January 2008 and June 2011, 48 consecutive patients who underwent surgery for 4-level CSM by a single surgeon (Shen HX) in our spine surgery center took part in the study. Inclusion criteria were (1) symptoms of cervical myelopathy and/or radiculopathy; (2) cervical spine X-ray, CT, and MRI showing intervertebral disc degeneration and herniation; and (3) cervical pathology at four consecutive levels. Exclusion criteria included ossification of the posterior longitudinal ligament (OPLL), rheumatoid arthritis, cerebral palsy, tumor, injury, destructive spondyloarthritis caused by hemodialysis, previous cervical surgery, thoracic spondylotic myelopathy, and lumbar spinal canal stenosis. Twenty seven (18 male and 9 female) between 47 and 76 years of age with compression restricted in the intervertebral spaces received ACDF (Group A). An additional 21 patients (13 male and 8 female) aged between 46 and 68 years, who had ventral spinal cord compression caused by pathology posterior to the vertebral body received SCF (Group B). The characteristics of the patients in the two groups are shown in Table 1.

### 2.2. Surgical Procedures

The surgical procedures performed on C3–7 have been described previously [10, 11]. For the patients in the ACDF group, the protrusive intervertebral discs and osteophytes were resected. An interbody fusion cage was implanted, and an anterior plate was used to improve the cervical spine alignment by providing multiple points of fixation. For the patients in the SCF group, C4 and C6 corpectomy was followed by resection of the osteophytes posterior to the C5 vertebral body. This was performed using extended decompression. An allograft bone graft or autogenous iliac bone graft was then implanted and fixed using an anterior plate.

**Figure 1:**
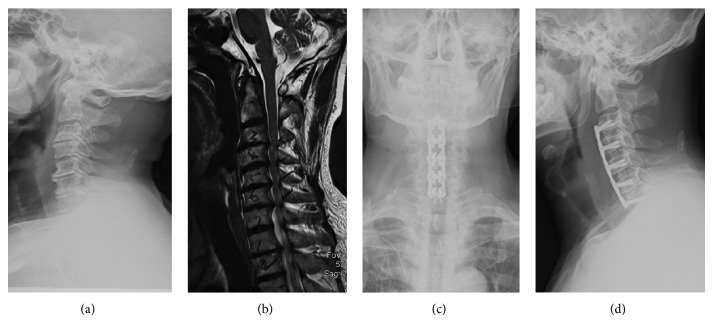
A 62-year-old male patient presented with a 6-month history of numbness in both hands which had become aggravated in the last month ago. This was accompanied by a month history of unsteadiness. Preoperative X-ray and MRI images ((a) and (b)) show loss of the cervical curvature with proliferation of osteophytes, and protrusions of C3/4 and C6/7 intervertebral discs. The compression of the spine was mainly restricted to the intervertebral space. No obvious compression was found on the posterior surface of the vertebral bodies. (c) and (d) show the anteroposterior and lateral images on neutral position of the cervical spine after ACDF surgery.

**Figure 2:**
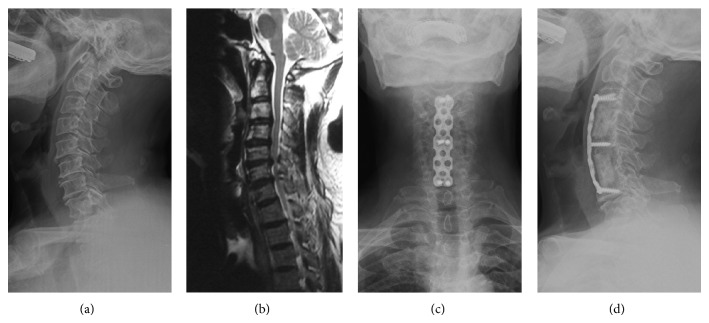
A 65-year-old male patient presented with a 2-year history of pain in the neck and shoulders accompanied by numbness in both hands. Preoperative X-ray and MRI images show proliferation of osteophytes, protrusions of C3/4 and C6/7, and spinal compression caused by hypertrophy of ligamentum flavum. (c) and (d) show the anteroposterior and lateral images on neutral position of the cervical spine after SCF surgery.

Slim-loc or Skyline anterior dynamic cervical plates (Johnson and Johnson Co., Depuy Spine Ltd., Ryhamn, MA) were used for fixation in both groups. X-rays were used to identify the affected segments during surgery. All patients were asked to wear cervical collar for 6 weeks. The patients were followed up at 6 weeks, 3, 12, 18, and 24 months postoperatively. Typical surgical outcomes are shown in Figures 1 and 2.

### 2.3. Postoperative Evaluation

**Figure 3:**
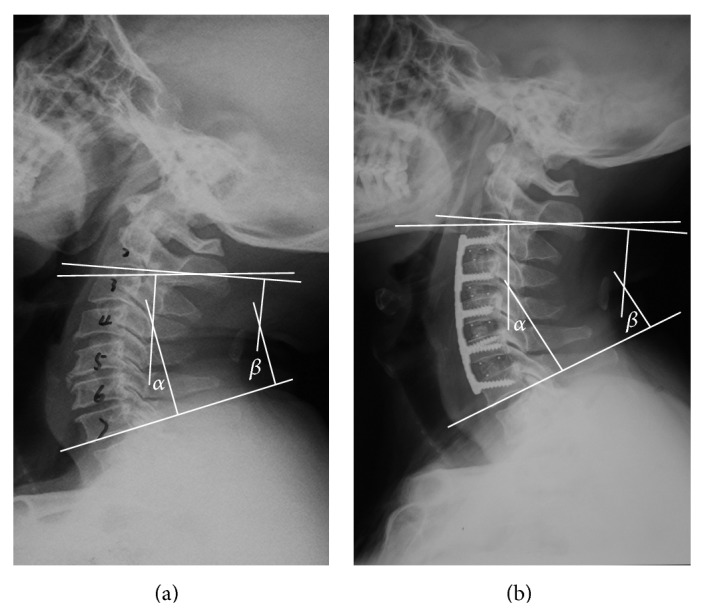
Measurement of Cobb's angle of C3–7 and C2–7 segments before (a) and after (b) surgery.

Perioperative data including patient characteristics, disease course, operation time, and blood loss volume were collected. Clinical efficacy was evaluated by preoperative and postoperative measurement of Japanese Orthopaedic Association (JOA) scores, neck disability index (NDI) score, and recovery rate of the JOA scores. Radiological outcomes included measurement of Cobb's angles for the operated segments (viz., C3–7 segments) and C2–7 segments. As shown in Figure 3, this was calculated as the angle between the superior border of the C3 vertebral body and the inferior border of the C7 or the angle between the inferior border of the C2 and the C7 vertebral body on neutral position. The criteria for osseous fusion were based on flexion and extension X-ray images showing that the spinous process distance was no more than 2 mm. Other criteria included absence of a radiolucent gap between the graft and the endplate; and/or demonstration of continuous bridging bony trabeculae at the graft-endplate interface [16–18]. Graft subsidence was defined as a >3 mm decrease in total vertical height of the two fused vertebral bodies measured on lateral cervical radiographs taken immediately postoperatively and at 1 day postoperatively [19].

The presence of the following postoperative complications was recorded: temporary hoarsenes, dysphagia, C5 nerve root palsy, cerebrospinal fluid leakage, pseudarthrosis, incision infection, graft subsidence, and graft dislocation. The incidence of dysphagia was defined as that solid or dry food gets stuck in the process of swallowing [20].

### 2.4. Statistical Analysis

Statistical analysis was undertaken using SPSS version 19.0 software (SPSS Inc., Chicago, IL, USA). Independent-sample *t*-tests were used to compare quantitative data including in the two groups. Paired-sample *t*-tests were used to compare the parameters before and after surgery. Pearson Chi-square tests were used to compare the incidence of postoperative complications. Values of *P* < 0.05 were considered statistical significant.

## 3. Results

### 3.1. Perioperative Conditions

As shown in Table 1, blood loss was significantly lower in ACDF group than in SCF group. However, no significant between group differences were found in sex, age, course of disease, follow-up, and operation time.

### 3.2. Clinical Parameters and Radiological Outcomes

As shown in Table 2, the JOA scores increased sharply postoperatively in patients in the ACDF and SCF groups. There were corresponding decreases in postoperative NDI scores in the two groups. There were no significant differences in JOA scores, NDI scores, and recovery rate of the JOA scores before and after the operation between the two groups.

Cobb's angle of the operated segments increased postoperatively in both groups. The Cobb's angle of the C2–7 segments increased from 10.7 ± 5.9 to 24.7 ± 6.3 in the ACDF group, and from 9.1 ± 5.9 to 18.1 ± 4.2 in the SCF group. No significant difference was found in Cobb's angles of the operated segments and C2–7 segments before the operation between the two groups. However, Cobb's angles of the operated segments and C2–7 segments after the operation were found significantly higher in the ACDF group than in the SCF group.

### 3.3. Postoperative Complications

Postoperative complications including temporary hoarseness (two patients), temporary dysphagia (six patients), and C5 nerve root palsy (three patients) were found in 11 patients in the ACDF group. In the SCF group, 15 patients developed postoperative complications (including one patient with temporary hoarseness, four with temporary dysphagia, two with C5 nerve root palsy, one with cerebrospinal fluid leakage, two with pseudarthrosis, one with incision infection, three with graft subsidence, and one with graft dislocation).

As shown in Table 3, postoperative complications were more common and more severe in the SCF group than in the ACDF group. In all patients, hoarseness, dysphagia, and C5 nerve root palsy improved after 2-week's conservative treatments and had resolved completely at the 6-week follow-up. In the SCF group, one patient with cerebrospinal fluid leakage recovered after 2 weeks by keeping the supine position, drainage and dressing change. Besides, complications associated with graft were found in six patients, including two with pseudarthrosis, three with graft subsidence, and one with graft dislocation. Because these patients did not have significative clinical symptoms, none of them received revision surgery, and they still have been followed.

## 4. Discussion

Cervical spondylotic myelopathy is a degenerative disease which mostly occurs in the elderly people. MRI images show that spinal cord compressions are mainly the result of protrusive intervertebral discs and osteophytes. The pathophysiologic features of CSM make anterior decompression the most effective surgical option.

Surgical treatment of multilevel CSM includes surgery through posterior, anterior and combined anterior and posterior surgical approaches. Both anterior and posterior approaches have been developed with the goal of decompressing the spinal cord and restoring the stability of the cervical spine. For some patients, with thickening of ligamentum flavum, in whom the compressions are mainly located at the dorsal spine, posterior laminectomy or laminoplasty is used to reduce the pressure. However, surgery through the posterior approach can result in progressive cervical kyphosis, C5 nerve root palsy, axial neck pain, segmental instability, and associated postoperative neurological deterioration, and may not completely reduce the pressure in patients with anterior compression (such as those with patients with intervertebral disc protrusion or osteophytosis), especially if there is co-existing kyphosis deformation.

Contiguous corpectomy and fusion was initially used for the treatment of 4-level CSM [21]. However, a study comparing three reconstructive techniques [22] demonstrated that ACCF was associated with high blood-loss, low fusion rate, a high incidence of postoperative complications, and relatively poor cervical lordosis restoration. On the basis of these findings ACCF is no longer considered the correct choice for treating multilevel CSM. A biomechanical study [23] demonstrated that long bone strut grafts and cervical plate which required a longer fixed force arm were needed for multilevel corpectomy. This resulted in a relatively high pressure being exerted on the rear end screw which increased the incidence of graft dislocation, dislodgement, and internal fixation failure [23]. Another biomechanical study which comparing SCF and CCF, reported that SCF provided better stability than CCF in terms of lateral bending, and found that fixation with six screws reduced peak screw pull-out force during axial rotation by 19% [24]. However, as the biomechanical stability of CCF is relatively poor, and as the technique is associated with severe postoperative complications such as nonunion, strut graft subsidence, and loss of lordosis, the procedure has been gradually replaced by other techniques.

Other researchers have suggested that the primary goal of treating 4-level CSM with ACDF should be decompression of the spinal cord, followed by realignment of the cervical spine, stabilization of the cervical spine, and correction of a cervical spinal deformity [10]. The same authors also noted that extended decompression with ACDF could not achieve complete reversal of compression in the posterior half of the vertebral bodies and they suggest that corpectomy should be performed in these cases. Other research [11] suggests that SCF requires less fusion interface than ACDF and shorter strut grafts, and for these reasons it may provide better stability without the need for fixation through a posterior approach. Therefore, SCF has the potential to achieve effective decompression with a lower risk of fusion failure.

The most common indication was cervical myelopathy, followed by a loss of cervical lordosis, cervical spinal cord compression, segmental instability, axial neck pain, radiculopathy, segmental instability, radiculomyelopathy, postlaminectomy deformity, failure of previous fusion, and central cord syndrome. For most cases, ACDF can achieve the purpose of thorough decompression. SCF is reserved for patients with ventral spinal cord compression caused by pathology posterior to the vertebral body and inaccessible via an extended discectomy [25].

### 4.1. Clinical Efficacy

ACDF and SCF both use an anterior approach to achieve direct decompression. Our analysis demonstrates that both techniques are associated with a similar level of efficacy, however, the blood loss volume was significantly lower in ACDF group as the procedure does not require the resection of vertebral body. As SCF may theoretically, could provide better decompression than ACDF, our own preference is to treat patients with ventral spinal cord compression caused by pathology posterior to the vertebral body with SCF, and to manage those with compression limited to the intervertebral spaces with ACDF. We believe that this management protocol may explain differences in the degree of compression between the two groups.

### 4.2. Cervical Spine Alignment

Previous workers have demonstrated that there is no significant difference in the postoperative realignment of the cervical spine between ACDF and HDF in the management of 3-level CSM [22]. However, the surgical failure rate increases significantly with the number of corpectomies that are required. Previous studies have also demonstrated that the failure rate caused by graft dislocation may be in the region of 50% to 71% when three contiguous corpectomies are performed [26, 27]. Our own findings demonstrate that ACDF results in better cervical spine alignment than SCF, suggesting that ACDF is better able to restore lordosis by providing fixation at multiple points, whereas graft subsidence, which could affect alignment of the cervical spine, is a general feature of SCF.

### 4.3. Postoperative Complications

A previous study [28] compared the incidence of complications (including graft subsidence, graft dislocation, hoarseness, dysphagia, C5 nerve root palsy, cerebrospinal fluid leakage, and incision infection) following the repair of 3-level CSM with three different reconstructive techniques using an anterior approach. The results suggested that ACDF was associated with the lowest incidence of pseudarthrosis and the highest incidence of laryngeal nerve-related complications. However, the highest overall incidence of complications was been found in CCF group, although no surgical treatment was required for most of the complications. Our analysis also showed that the incidence of complications in patients with 4-level CSM was significantly higher than that reported for 3-level CSM. In our series of cases complications in the ACDF group mainly included temporary hoarseness, dysphagia, and C5 nerve root palsy. Moreover, the incidence of severe complications such as incision infection, graft dislocation, and graft subsidence were significantly lower than in the SCF group. With corpectomies in the SCF group, long strut grafts and plate fixations create long lever arms that stress the caudal screws, making patients vulnerable to graft migration, displacement, and subsidence and instrumentation failure. These findings suggest that ACDF may be associated with a better safety profile than SCF.

### 4.4. Adjacent Segment Degeneration

For patients with multilevel CSM, the incidence of postoperative adjacent segment degeneration (ASD) is the most important concern for researchers. The incidence rate of ASD after anterior cervical fusion has been estimated at about 2.9% [29]. Previous studies suggest that the biomechanical changes of the cervical spine involved in the fusion of multiple segments may increase the mobility of adjacent segments, which in turn, increases compression on the intervertebral discs and accelerates discdegeneration. Thus, the incidence of ASD has been shown to increase with the number of fused segments [30–32]. However, other research suggests that ASD is caused by the dual action of natural and accelerated degeneration of adjacent segments [29, 33]. In accordance with this hypothesis, a systematic review identified pre-existent adjacent segment degeneration as the main determinant of ASD [34]. Other studies have shown that the incidence of ASD is significantly lower after anterior cervical fusion undertaken for multilevel disease than for single-level disease. This may be explained by the incorporation of the high-risk (C5-C6, C6-C7) and intermediate-risk (C3-C4, C4-C5) levels into the fusion construct that occur adjacent to levels at low risk for new disease (C2-C3, C7–T1) [29]. In our series of patients there was no evidence of ASD after a follow-up of 2 years or more.

Limitations to the present study include the retrospective design, relatively short follow-up time, small sample size, and compression differences between the two groups which could theoretically bias the results. However, to date no study has investigated differences between ACDF and SCF. This is, therefore, the first study to demonstrate that ACDF involves less intraoperative blood loss, better cervical spine alignment, and fewer postoperative complications than SCF. Randomized controlled trials with larger sample sizes and longer follow-up times are needed to further investigate the differences between these two reconstructive techniques.

## Figures and Tables

**Table 1:** Characteristics of the patients.

	ACDF group (*N* = 27)	SCF group (*N* = 21)	*P* value
Age (year)	57.3 ± 8.0	56.7 ± 6.3	0.259
Gender (male/female)	18/9	13/8	0.518
Disease course (month)	11.2 ± 13.7	10.7 ± 8.1	0.121
Duration of follow-up (month)	34.7 ± 6.9	33.1 ± 8.4	0.156
Operated segments	C3–7	C3–7	
Operation time (minute)	152.6 ± 24.2	148.3 ± 25.9	0.733
Blood loss (mL)	161.1 ± 42.4	292.9 ± 67.6	0.027^*^

Values shown are means ± SD. ACDF: anterior cervical discectomy and fusion; SCF: “skip” corpectomy and fusion. ^*^ *P* < 0.05.

**Table 2:** Clinical parameters and radiological outcomes.

	ACDF group (*N* = 27)	SCF group (*N* = 21)	*P* value
JOA score			
Preoperative	8.5 ± 1.3	8.4 ± 1.2	0.697
End of the follow-up	12.7 ± 1.4^**^	13.9 ± 1.6^**^	0.965
Increase (%)	49.9 ± 15.3	64.1 ± 16.5	0.690
NDI score			
Preoperative	35.2 ± 3.3	34.0 ± 3.7	0.652
End of the follow-up	14.8 ± 4.0^**^	13.3 ± 3.6^**^	0.524
Cobb's angle of C3–7 (°)			
Preoperative	8.5 ± 4.9	8.1 ± 5.5	0.510
End of the follow-up	21.7 ± 6.5^**^	15.8 ± 4.4^**^	0.025^*^
Cobb's angle of C2–7 (°)			
Preoperative	10.7 ± 5.9	9.1 ± 5.9	0.988
End of the follow-up	24.7 ± 6.3^**^	18.1 ± 4.2^**^	0.027^*^

Values shown are means ± SD. ACDF: anterior cervical discectomy and fusion; SCF: “skip” corpectomy and fusion. JOA: Japanese Orthopaedic Association; NDI: neck disability index. ^*^ *P* < 0.05; ^**^relative to values before surgery.

**Table 3:** Complications.

Complication	Number (%) of patients		
	ACDF group (*N* = 27)	SCF group (*N* = 21)	*P* value
Temporary hoarseness	2 (7.4%)	1 (4.76%)	0.707
Temporary dysphagia	6 (22.2%)	4 (19.1%)	0.788
C5 nerve root palsy	3 (11.1%)	2 (9.5%)	0.858
Cerebrospinal fluid leakage	0	1 (4.8%)	0.252
Pseudarthrosis	0	2 (9.5%)	0.101
Incision infection	0	1 (4.8%)	0.252
Graft subsidence	0	3 (14.3%)	0.043^*^
Graft dislocation	0	1 (4.8%)	0.252

Total	11 (40.7%)	15 (71.4%)	0.034^*^

ACDF: anterior cervical discectomy and fusion; SCF: “skip” corpectomy and fusion. ^*^ *P* < 0.05.
